# Imaging of genomic loci in *Trypanosoma brucei* using an optimised LacO-LacI system

**DOI:** 10.1016/j.molbiopara.2023.111598

**Published:** 2023-11-03

**Authors:** James Budzak, Ione Goodwin, Calvin Tiengwe, Gloria Rudenko

**Affiliations:** Department of Life Sciences, Sir Alexander Fleming Building, Imperial College London, South Kensington, London SW7 2AZ, UK

**Keywords:** Trypanosoma brucei, Nuclear architecture, LacO-LacI, Microscopy, Gene expression

## Abstract

Visualisation of genomic loci by microscopy is essential for understanding nuclear organisation, particularly at the single cell level. One powerful technique for studying the positioning of genomic loci is through the Lac Operator-Lac Repressor (LacO-LacI) system, in which LacO repeats introduced into a specific genomic locus can be visualised through expression of a LacI-protein fused to a fluorescent tag. First utilised in *Trypanosoma brucei* over 20 years ago, we have now optimised this system with short, stabilised LacO repeats of less than 2 kb paired with a constitutively expressed mNeongreen::LacI fusion protein to facilitate visualisation of genomic loci. We demonstrate the compatibility of this system with super-resolution microscopy and propose its suitability for multiplexing with inducible RNAi or protein over expression which will allow analysis of nuclear organisation after perturbation of gene expression.

*Trypanosoma brucei* is an important human pathogen and a good model organism for the study of fundamental concepts underpinning gene expression and nuclear organisation [1,2]. Several important techniques adapted to *T. brucei* have been used to better understand nuclear architecture including Hi-C, DNA/RNA-FISH and the LacO-LacI system [3–8]. The LacO-LacI system was first introduced to *T. brucei* by Navarro and Gull in 2001. Using this system, these authors were able to conclusively show for the first time that the active bloodstream form expression site (BES) is located in a specific nuclear sub-compartment, termed the Expression Site Body (ESB) [8]. This observation revolutionised our understanding of how singular expression of the major surface antigen in *T. brucei* (Variant Surface Glycoprotein) is achieved. The LacO-LacI system was subsequently used in other studies of this trypanosome but has not been widely adopted [9,10]. In these previous studies, 256xLacO repeats were inserted into a genomic locus and their visualisation was achieved through inducible expression of an eGFP:: LacI fusion protein which binds to the LacO repeats. Although functional, in that system the presence of the LacO repeats can be cytotoxic and inducible expression of LacI fusion proteins can result in undesirable heterogeneity in expression levels [8,10]. We therefore sought to mitigate these issues by optimising the LacO-LacI system for more tractable and reproducible use in marking diverse genomic loci in this trypanosome.

Initially, the pSmOx plasmid which contains both a T7 RNA polymerase and tetracycline repressor (TetR) gene was modified such that an mNeongreen (mNG) LacI fusion protein with a C-terminal SV40 nuclear localisation signal was inserted downstream of the TetR gene (Fig. 1A). When linearised, the plasmid can be targeted to integrate upstream of the first β-tubulin gene within the tubulin array, leading to the constitutive expression of the fusion protein (Fig. 1A; [11]). Following transfection into wild type bloodstream form (BSF) *T. brucei* Lister 427 cells, fluorescence microscopy analysis of this cell line (pSmOx-mNG::LacI) revealed near uniform cell-to-cell mNG::LacI signal (Fig. 1B), for the full-length 66.5 kDa fusion protein (Fig. 1C).

Next, the pDex577 plasmid [12] was modified to allow targeting of LacO repeats of variable length into the 177 bp repeats of the *T. brucei* genome (present in mini chromosomes and intermediate chromosomes; [13]). The LacO repeats we used here were modified such that a random 10 nt spacer was inserted between each repeat to increase sequence degeneracy, which will likely help prevent loss of the LacO repeat sequence [14]. Interestingly, it has been reported that shortening of the LacO repeats (from x256 to x64 repeats) may enhance sensitivity for detecting LacO-LacI foci [15]. For this reason, we tested LacO repeat lengths of 50x, 120x, 162x or 240x (with sizes ranging from 1.8 kb to 9.6 kb) integrated into the 177 bp repeats of the pSmOx-mNG::LacI cell line. Analysis of the resulting cell lines revealed that increasing the LacO repeat length in this region caused a severe growth defect (Fig. 2C). However, only a marginal growth defect was observed in the 177 bp-LacOx50 cell line compared to the pSmOx-mNG::LacI cells without integrated repeats (Fig. 2C). This effect did not appear to be due to insertion of large plasmids into the 177 bp repeats, as integration of the pDex577 plasmid (7.5 kb) into the same region did not result in a noticeable growth defect. Importantly, imaging of all 177 bp-LacO cell lines by fluorescence microscopy showed clear detection of a LacO-LacI focus (92 % of cells) even when using only 50x repeats, indicating that shorter LacO repeats do not compromise the sensitivity of detection (Fig. 1E and Fig. 1F). Furthermore, we observed a decrease in cell-to-cell uniformity in LacO-LacI foci detection when the number of LacO repeats in the 177 bp repeat region was increased (Fig. 1F).

We subsequently determined if the 50xLacO repeat sequence could be visualised by microscopy when integrated into different genomic loci in the presence of the mNG::LacI fusion protein. To target 50xLacO repeats to BES1, plasmids were generated in which the 50xLacO repeats were placed upstream of the BES1 promoter (homologous regions amplified from TAR40 DNA and H25N7 bacterial artificial chromosome DNA; [16,17]) with a downstream hygromycin drug selection marker (Fig. 2A). Transfection of this plasmid results in exchange of the endogenous BES1 promoter, integrating the LacO repeat sequence approximately 160 bp upstream of the core BES promoter. To target the LacOx50 repeats to the rDNA spacer, the homologous region of an rDNA spacer targeting plasmid [18] was used to integrate the LacO sequence upstream of an endogenous rDNA promoter (Fig. 2B).

We confirmed that the mNG::LacI fusion proteins were being targeted to the correct nuclear compartment by co-localisation of the fusion protein with protein markers associated with the active BES1 or rDNA. For the BES1-LacOx50 cell line, the second largest subunit of RNA Pol I (RPA2) was endogenously tagged with a TdTomato fluorescence protein [1]. In addition to transcribing the rDNA, RNA Pol I also transcribes the active BES in *T. brucei* in the ESB. The ESB is a discrete extra-nucleolar focus which is enriched in RNA Pol I [8,19]. As expected, we found that the LacO-LacI focus in the BES1-LacOx50 cell line was either co-localised with, or adjacent to the ESB, with a LacO-LacI focus detectable in 92 % of cells (Fig. 2A and Fig. 2D). Furthermore, we did not observe a decrease in the percentage of cells with an ESB after insertion of the LacOx50 repeat sequence upstream of the BES1 promoter (Fig. 2A). This suggests that BES1 transcription is not affected by the presence of the LacOx50 repeats, as disruption of BES transcription has been shown to result in a decrease in the percentage of cells with an ESB [1,19,20]. In the rDNA-LacOx50 cell line, correct targeting of the mNG:: LacI fusion protein to rDNA was verified by performing co-localisation with the nucleolar marker L1C6 [1,20–22] (Fig. 2B). In 84 % of cells, a distinct mNG::LacI focus was detectable at the nucleolar periphery (Figs. 2B and 2D). Importantly, a high percentage of cells with LacO-LacI foci were detected regardless of the target integration site for the LacO repeats with comparable sensitivity between rDNA, the BES1 promoter region and 177 bp repeats. In addition, no significant growth defect was observed in the BES1-LacOx50 or rDNA-LacOx50 cell lines relative to the parental cell line without LacO repeats (Fig. 2C).

To test the compatibility of this system with other microscopy applications, the BES1-LacOx50 plasmid was integrated into a cell line expressing mNG::LacI and an endogenously tagged Halo::RPA2 fusion protein [1]. Structured illumination super-resolution microscopy (SR-SIM) on these cells showed that a LacO-LacI focus was detectable in 90 % of cells and was co-localised with the ESB (Fig. 2D and 2E). This result demonstrates that this LacO-LacI system has sufficient signal-to-noise and photostability to be used for super-resolution imaging.

In conclusion, we have developed a simplified and improved LacO-LacI system for imaging of individual genomic loci in BSF *T. brucei*. Although dCas9 based imaging systems have recently been developed, robust imaging of single copy, non-repetitive loci using dCas9 is challenging [23] and no such system has currently been described for kinetoplastids. Therefore, the LacO-LacI system remains a useful tool for investigating nuclear organisation by microscopy-based imaging in *T. brucei*. The optimised system we present allows visualisation of short LacO repeats (x50, 1.8 kb) via a constitutively expressed mNG::LacI fusion protein which minimises the impact on cell viability. In addition, we observed clear detectable LacO-LacI foci when visualising genomic loci with either standard or super-resolution microscopy. This system is also compatible with subsequent genetic modification of BSF *T. brucei* for inducible RNAi or protein overexpression, providing a sensitive method for visualising genomic loci following perturbations to nuclear architecture and/or gene expression. Furthermore, PCR based tagging methods could potentially be used to integrate LacO repeats into specific genomic loci of interest [24]. Some of the fundamental aspects of the system are also likely to be transferable to other kinetoplastids for interrogating species-specific genome biology. All plasmids generated in this study (plus additional LacO plasmids; listed in Table 1) are freely available from the corresponding author and can be obtained from Addgene (#83397).

## Figures and Tables

**Fig. 1:**
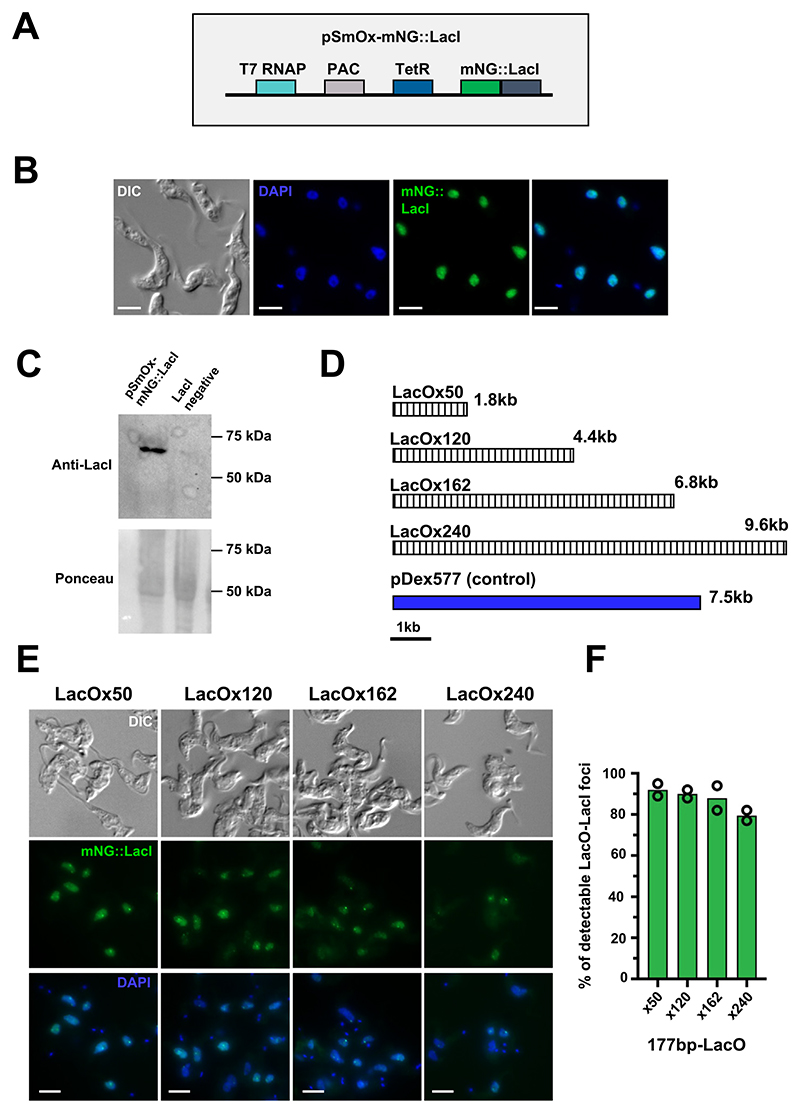
Optimisation of a LacO-LacI system in *Trypanosoma brucei*. **A)** Schematic showing the organisation of a modified pSmOx2 plasmid (pSmOx-mNG::LacI) after linearisation with *Hin*dIII. The plasmid contains a T7 RNA polymerase (T7 RNAP), puromycin N-acetyl-transferase (PAC), tetracycline repressor (TetR) and mNeongreen::Lac repressor (mNG::LacI) genes. **B)** Representative fluorescence microscopy images of cells constitutively expressing mNG::LacI (green). **C)** Western blot analysis of cell line shown in **(B)** using an anti-LacI antibody (clone 9A5, Sigma-Aldrich) to detect the mNG::LacI fusion protein (66.5 kDa). 1 × 10^7^ cell equivalents were loaded per lane. **D)** Schematic showing different LacO repeat lengths integrated into the genome using 177 bp repeat targeting sequences. The number of LacO repeats and a 1 kb scale bar are shown. The pDex577 fusion protein plasmid was used as a control for integration of large constructs into the minichromosome. **E)** Representative fluorescence microscopy images of cells expressing mNG::LacI (green) with either LacOx50, LacOx120, LacOx162 or LacOx240 integrated into a 177 bp repeat. For microscopy images, DNA is stained with DAPI (blue). Samples were fixed in 4 % PFA and imaged with a Zeiss AxioImager M2 using a Hamamatsu ORCA-Flash4 camera. A full description of the imaging system and sample preparation can be found in [1]. All scale bars are 5 µm. **F)** Quantification of the percentage of cells with detectable LacO-LacI foci from the cell lines shown in (E). At least 100 G1 cells were quantified from two biological replicates.

**Fig. 2:**
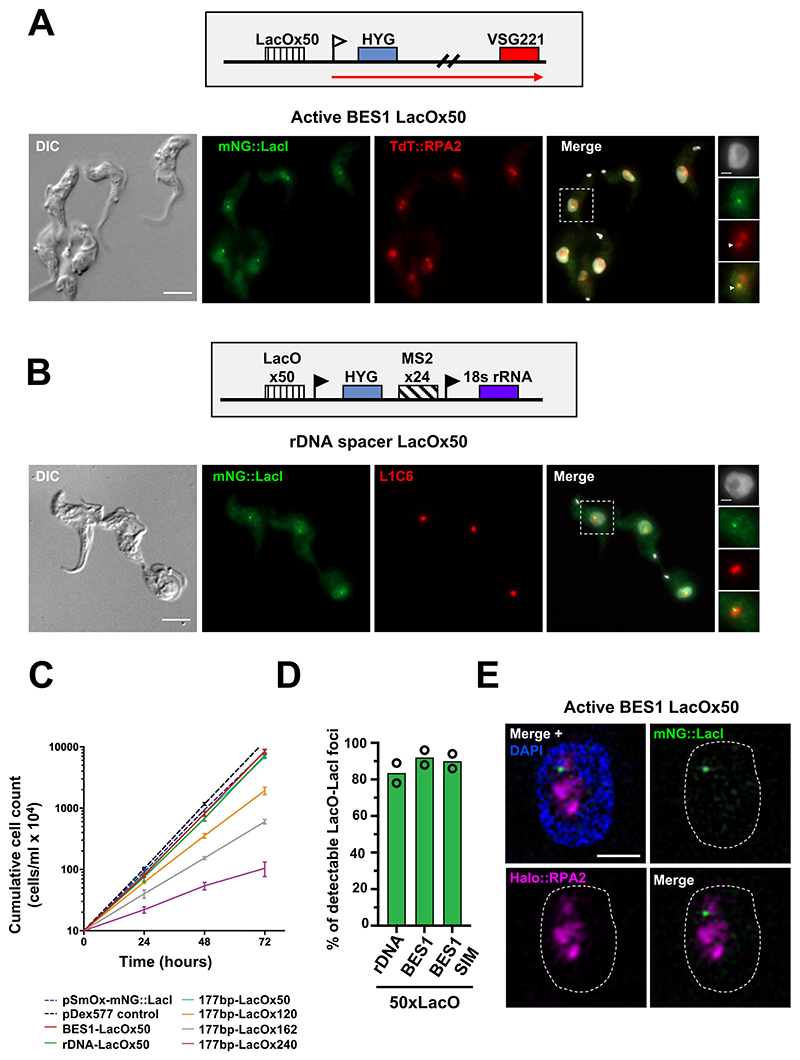
Optimised LacO-LacI system with 50xLacO repeats enables visualisation of different genomic loci in BSF *T. brucei* and is suitable for super-resolution imaging. **A)** A schematic of the modified BES1 promoter after transfection of the BES1-LacOx50 plasmid is shown with genes indicated by boxes, the BES1 promoter with a white flag and active transcription noted by a red arrow. Representative fluorescence microscopy images of cells expressing mNG::LacI (green) and TdT::RPA2 (red) with 50xLacO repeats integrated immediately upstream of the active BES1 promoter. **B)** Representative fluorescence microscopy images of cells expressing mNG::LacI (green) with 50xLacO repeats integrated into the rDNA spacer. The nucleolus is stained with an L1C6 antibody (red). A schematic of the modified rDNA spacer is shown, genes are indicated with boxes and rDNA promoters are indicated with black flags. **C)** Cumulative growth curve of the BES1-LacOx50, rDNA-LacOx50 and all 177 bp-LacO cell lines. Cells were seeded to an estimated density of 1×10^5^/ml, counted every 24 h in technical triplicates and diluted daily. **D)** Quantification of the percentage of 1K1N cells with detectable LacO-LacI foci in which LacO repeats are integrated in the rDNA spacer (rDNA) and the active BES1 promoter region (BES1). Data shown are averages from two biological replicates with a minimum of 100 cells per replicate with the exception of the BES1 super-resolution structured illumination microscopy (SR-SIM) quantification (50 1K1N cells from two biological replicates). **E)** SR-SIM imaging of a cell line expressing Halo::RPA2 (magenta) and mNG::LacI (green) with 50xLacO repeats integrated upstream of the active BES1 promoter. Super-resolution imaging was performed as described in [1] using an Elyra PS.1 microscope with a sCMOS PCO Edge Camera. For microscopy images, the nucleus is delineated with a dashed line and DNA is stained with DAPI. Scale bars are 5 µm and for SR-SIM and inset images scale bars are 1 µm. All images shown are maximum intensity projections of z-stacks (except for the DIC channel). Immunofluorescence microscopy imaging, SR-SIM imaging and Halo tag labelling were performed as described in [1].

**Table 1:** Overview of plasmids used to insert LacO repeats into different genomic loci. Prior to transfection, LacO repeat plasmids targeting the 177 bp repeats and rDNA spacer are linearised with *Not*I while LacO repeat plasmids targeting the BES1 promoter region are linearised with *Avr*II and *Kpn*I. The pSMOX-mNG:: LacI plasmid is linearised with *Hin*dIII. All plasmids listed here are available from the corresponding author and can be obtained from Addgene (deposit #83397).

Plasmid name	Integration site	Drug selection marker
p177bp-LacOx50-Hyg	177 bp repeats	Hygromycin
p177bp-LacOx120-Hyg	177 bp repeats	Hygromycin
p177bp-LacOx162-Hyg	177 bp repeats	Hygromycin
p177bp-LacOx240-Phleo	177 bp repeats	Phleomycin
p177bp-LacOx50–24xMS2-Hyg	177 bp repeats	Hygromycin
p177bp-LacOx120–24xMS2-Hyg	177 bp repeats	Hygromycin
prDNA spacer-LacOx50–24xMS2-Hyg	rDNA spacer	Hygromycin
prDNA spacer-LacOx120–24xMS2-Hyg	rDNA spacer	Hygromycin
pBES1-LacOx50-blast	Upstream of BES1 promoter	Blasticidin
pBES1-LacOx50-Hyg	Upstream of BES1 promoter	Hygromycin
pBES1-LacOx50-mCh-bla	Upstream of BES1 promoter	Blasticidin
pSmOx-mNG∷LacI-V3	Tubulin locus	Puromycin
